# Dexmedetomidine Versus Propofol for Patients With Sepsis Requiring Mechanical Ventilation: A Systematic Review and Meta-Analysis

**DOI:** 10.3389/fphar.2021.717023

**Published:** 2021-10-14

**Authors:** Po Huang, Xiangchun Zheng, Zhi Liu, Xiaolei Fang

**Affiliations:** Beijing Dongfang Hospital, Beijing University of Traditional Chinese Medicine, Beijing, China

**Keywords:** sepsis, mechanical ventilation, dexmedetomidine, propofol, meta-analysis

## Abstract

**Purpose:** This meta-analysis was performed to access the influence of dexmedetomidine versus propofol for adult patients with sepsis undergoing mechanical ventilation.

**Materials and Methods:** NCBI PUBMED, Cochrane Library, Embase, China National Knowledge Internet (CNKI), and China Biological Medicine (CBM) were searched. Revman 5.3 and Stata software (version 12.0, Stata Corp LP, College Station, TX, United States) were used for meta-analysis.

**Results:** Fifteen studies were included, and the data from the included studies were incorporated into the meta-analysis. Also, the result shows that compared with propofol, dexmedetomidine does not reduce 28-day mortality [risk ratios (RR) =0.97, 95% confidence interval (CI) =0.83–1.13, *p* = 0.70]. However, our analysis found that dexmedetomidine could reduce intensive care unit (ICU) stays {standard mean difference (SMD): −0.15; 95% CI: [−0.30–(−0.01)], *p* = 0.03}, duration of mechanical ventilation {SMD: −0.22; 95% CI: [−0.44–(−0.01)], *p* = 0.043}, sequential organ failure assessment (SOFA) {SMD: −0.41; 95% CI: [−0.73–(−0.09)], *p* = 0.013}, levels of interleukin-6 (IL-6) at 24 h (SMD: −2.53; 95% CI: −5.30-0.24, *p* = 0.074), and levels of CK-MB at 72 h {SMD: −0.45; 95% CI: [−0.83–(−0.08)], *p* = 0.017}.

**Conclusions:** This meta-analysis (MA) suggests that in terms of 28-day mortality, sepsis patients with the treatment of dexmedetomidine did not differ from those who received propofol. In addition, more high-quality trials are needed to confirm these findings.

**Systematic Review Registration:** https://www.crd.york.ac.uk/prospero/#recordDetails, identifier CRD42021249780.

## Introduction

According to the recent reports, approximately 21% of septic patients required mechanical ventilation in the United States (Rashmi et al., 2018). As we all know, patients with prolonged mechanical ventilation suffer from higher mortality and hospital costs (Goligher Ewan et al., 2018; Louise, 2020). Besides, appropriate sedation measures are necessary for patients with sepsis undergoing mechanical ventilation because these measures are taken to avoid a series of adverse reactions caused by mechanical ventilation, including anxiety and delirium (Ohta et al., 2020). It had been reported that the early deep sedation was associated with the increased ventilation duration and the mortality (Yahya et al., 2012). Given this, the guidelines recommend dexmedetomidine or propofol to be applied to adult patients receiving mechanical ventilation for targeting mild sedation (Shankar et al., 2016).

As a potent α2 agonist with antianxiety, sedative, analgesic, and sympathetic properties, dexmedetomidine (DEX) is widely used in ICU for mild sedation (Flieller Lauren et al., 2019). Propofol, chemically known as 2,6-diisopropylphenol, is a type of rapid and short-acting intravenous anesthetic commonly used clinically for induction of anesthesia, maintenance of anesthesia, and sedation in critical patients in ICU. It has the advantages of fast onset of anesthesia induction, rapid recovery and perfect functional recovery, and low incidence of postoperative nausea and vomiting (Doi et al., 2020). However, both the drugs have side effects, and there are differences in wake and inflammation between them (Li et al., 2021). Besides, it still remains unknown whether these two drugs affect the research outcomes on mechanical ventilation for adult patients with sepsis.

Recently, some randomized controlled trials have been conducted with respect to the comparison of DEX and propofol in the treatment of sepsis. However, there is still much controversy in the effects of DEX and propofol on mortality, ICU stays, and incidence of adverse events. Against this background, it is necessary to systematically evaluate the efficacy and safety of DEX and propofol in the treatment of sepsis with mechanical ventilation so as to provide evidence-based evidence.

## Methods

The preferred reporting items for systematic review and a meta-analysis (PRISMA) statement (Nikola et al., 2013) have provided the details of meta-analysis, and all the reviews should be conducted according to the content of PRISMA. Therefore, our meta-analysis was performed based on the recommendations and checklist from PRISMA.

### Search Strategy

We searched the relevant studies from Pubmed, Cochrane Library, Embase, CNKI, and CBM from their inception to May 2021.

### Eligibility Criteria of Original Studies

Diagnostic criteria of sepsis: infection combined with SOFA ≥2.

Inclusion criteria: the original studies we selected should meet PICOS as follows: 1) participants: mechanically ventilated adult patients with sepsis, regardless of the country, region, gender, or nationality; 2) interventions: dexmedetomidine with continuous intravenous pumping; 3) control: propofol with continuous intravenous pumping; 4) outcomes: primary outcome mainly refers to the 28-day mortality; secondary outcomes cover ICU stays, duration of mechanical ventilation, incidence of adverse events, sequential organ failure assessment (SOFA), levels of interleukin-6 (IL-6) at 24 h, and levels of CK-MB at 72 h; 5) study design: the study was designed as the randomized controlled trial (RCT).

Exclusion criteria: the exclusion criteria were a supplement to the inclusion criteria, and those studies which meet the following conditions will be excluded: 1) the duplicate publications, 2) the participants were children, 3) the diagnostic criteria of sepsis were ambiguous, and 4) the data cannot be used or their source is unknown.

### Study Selection

Two reviewers independently screen the studies according to the preset criteria for inclusion and exclusion, in which the title and the abstract are the main references. Meanwhile, the full text will be checked if necessary. Once the two independent reviewers diverge in the definition of the included study, the third independent reviewer will intervene in time and actively resolve within the group. If the diverge still cannot be solved, the agreement will be reached by consensus.

### Data Extraction and Quality Assessment

Based on pre-planned results, the related information from the identified studies is extracted by two reviewers independently. For example, this information, including first author, year of publication, sample size, interventions, controls, and results, should be recorded in detail and edited into a table form. Once the two independent reviewers diverge in the definition of the included study, the third independent reviewer intervenes in time and actively resolves within the group. If it still cannot be solved, the agreement will be reached by consensus.

In addition, the quality of the included studies is assessed by two reviewers independently. Also, the Jadad Scale is used to evaluate the quality of randomized controlled trials. According to the principle, 1–4 indicates a low-quality study and 5–7 indicates a high-quality study, and the maximum of Jadad score is 7.

### Data Synthesis

Revman 5.3 and Stata 12.0 software (Stata Corp LP, College Station, TX, United States) are used to analyze the following information: 28-day mortality, ICU stays, duration of mechanical ventilation, incidence of adverse events, SOFA, levels of IL-6 at 24 h, and levels of CK-MB at 72 h. Based on the recommendations of Cochrane Handbook of Systematic Reviews, risk ratios (RRs) and 95% confidence intervals (CIs) are employed to evaluate the dichotomous results. For continuous results, the standard mean difference (SMD) and its 95% CI are selected. Heterogeneity between studies is evaluated by the I^2^ test. The fixed-effect model is applied if there is no or low heterogeneity (I^2^ ≤ 25%). Otherwise, the randomized effect model will be employed if there exists heterogeneity (I^2^ > 25%). Also, publication bias is also evaluated (the number of studies ≥10 in one outcome).

### Subgroup Analysis

Subgroup analysis was conducted in the outcome of 28-day mortality based on the evidence covering studies published in English versus non-English, high Jadad score (≥5) versus low Jadad score, and high dose of dexmedetomidine (≥0.5 μg/kg/h) versus low dose of dexmedetomidine.

### PROSPERO Registration

Before the meta-analysis was formally conducted, we registered the topics, the inclusion and exclusion criteria, the outcomes, and the statistical analysis methods on PROSPERO so as to show the whole process of the meta-analysis in a more open and transparent way. The details of PROSPERO registration could refer to https://www.crd.york.ac.uk/prospero/#recordDetails.

## Results

### Included Studies

**FIGURE 1:**
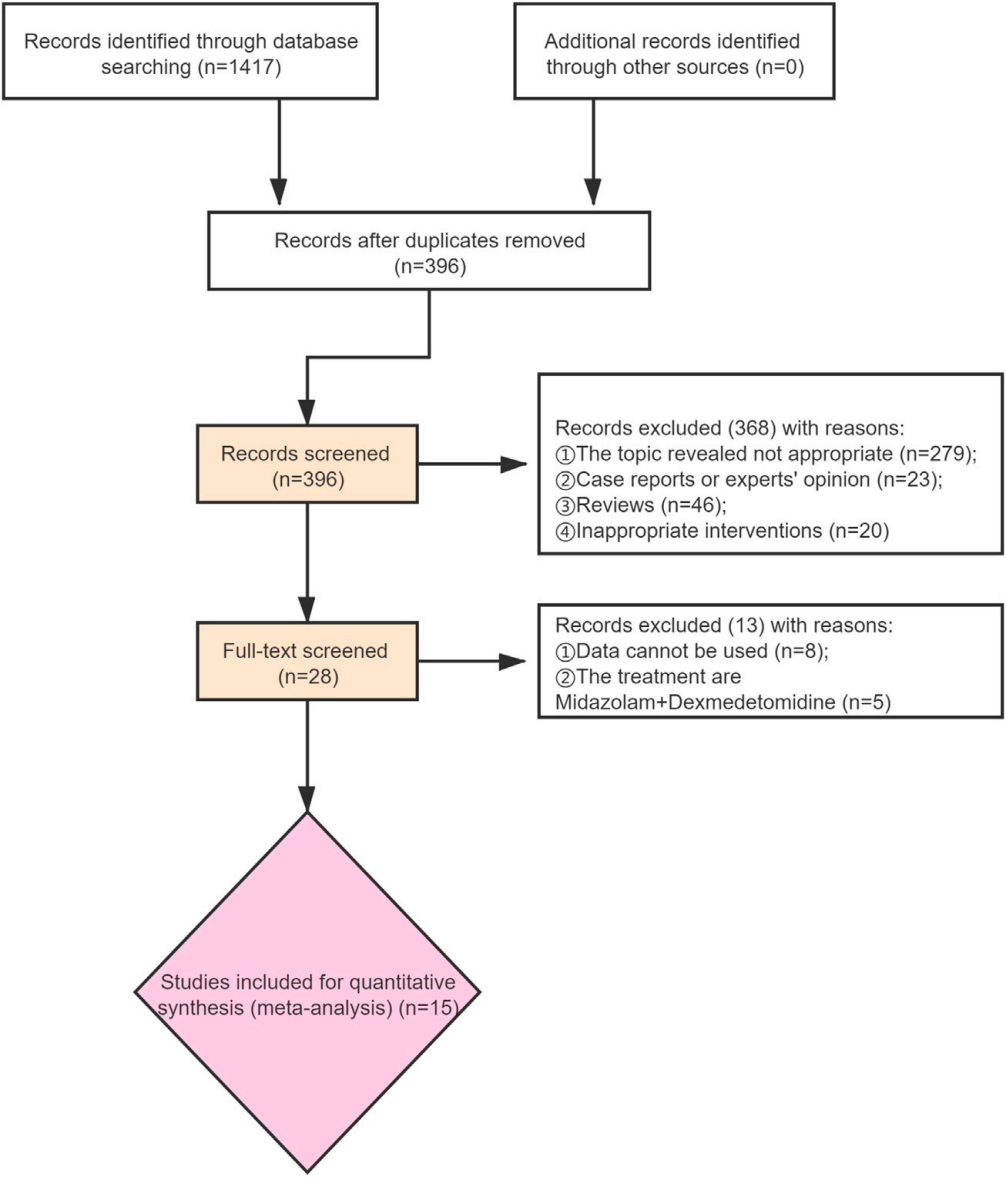
Flow chart of included studies selection.

A total of 1,417 records were identified, and 396 records were obtained after removing duplicate publications. After that, 368 studies were removed by screening the titles and abstracts. This means that 28 studies have been further screened through reading the full text. It was found among them that the data of nine studies cannot be accessible, and the intervention measures of five studies do not meet the inclusion criteria. In total, 1,871 participants in 15 studies were included (Figure 1). The details of included studies are shown in Table 1.

**TABLE 1:** The characteristics of the included studies.

Study	No. of participants	Intervention		Outcomes
		Experimental group	Control group	
Meng et al. (2014)	N = 40 (T = 20; C = 20)	Dexmedetomidine	Propofol	28-day mortality, ICU stays, Duration of mechanical ventilation, levels of IL-6 at 24 h
Guo et al. (2016)	N = 30 (T = 14; C = 16)	Dexmedetomidine	Propofol	28-day mortality, ICU stays, Duration of mechanical ventilation
Lei et al, 2016)	N = 58 (T = 29; C = 29)	Dexmedetomidine	Propofol	28-day mortality, ICU stays, levels of CK-MB at 72 h
Zhou (2017)	N = 80 (T = 40; C = 40)	Dexmedetomidine	Propofol	28-day mortality, ICU stays, levels of CK-MB at 72 h
Kawazoe et al. (2017)	N = 201 (T = 100; C = 101)	Dexmedetomidine	Propofol	28-day mortality, Duration of mechanical ventilation, Incidence of adverse events
Ding et al. (2019)	N = 282 (T = 131; C = 152)	Dexmedetomidine	Propofol	ICU stays, Incidence of adverse events, levels of CK-MB at 72 h
Liu JQ et al. (2019)	N = 200 (T = 100; C = 100)	Dexmedetomidine	Propofol	28-day mortality, Duration of mechanical ventilation
Wang QS et al. (2019)	N = 101 (T = 42; C = 59)	Dexmedetomidine	Propofol	28-day mortality, ICU stays, Incidence of adverse events, levels of IL-6 at 24 h
Wang YF et al. (2019)	N = 63 (T = 31; C = 32)	Dexmedetomidine	Propofol	28-day mortality, ICU stays, Incidence of adverse events
Liu SC et al. (2019)	N = 63 (T = 31; C = 32)	Dexmedetomidine	Propofol	Incidence of adverse events, SOFA, levels of IL-6 at 24 h
Xu (2019)	N = 50 (T = 25; C = 25)	Dexmedetomidine	Propofol	28-day mortality, ICU stays, Duration of mechanical ventilation, levels of IL-6 at 24 h
Cai et al. (2019)	N = 60 (T = 30; C = 30)	Dexmedetomidine	Propofol	28-day mortality, ICU stays, Duration of mechanical ventilation, Incidence of adverse events, SOFA
Liu Z et al. (2020)	N = 102 (T = 51; C = 51)	Dexmedetomidine	Propofol	28-day mortality, ICU stays, Duration of mechanical ventilation, SOFA
Wei GW et al. (2020)	N = 119 (T = 60; C = 59)	Dexmedetomidine	Propofol	28-day mortality, Incidence of adverse events

### Quality Assessment of the Included Studies

The quality of the included studies was evaluated by the Jadad Scale, which covers the generation of random sequences, allocation concealment, the blinding method, and reasons for withdrawal or dropout. The result showed that most of the included studies obtained low scores, among which there were only four studies with high scores (Kawazoe et al., 2017; Ding et al., 2019; Liu et al., 2020; Zhan et al., 2020). The details are demonstrated in Supplementary Table S1.

### Primary Outcome

**FIGURE 2:**
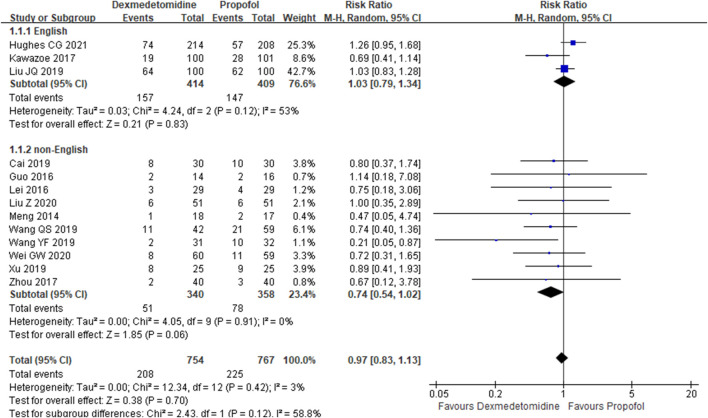
Forest plot of 28-day mortality.

**FIGURE 3:**
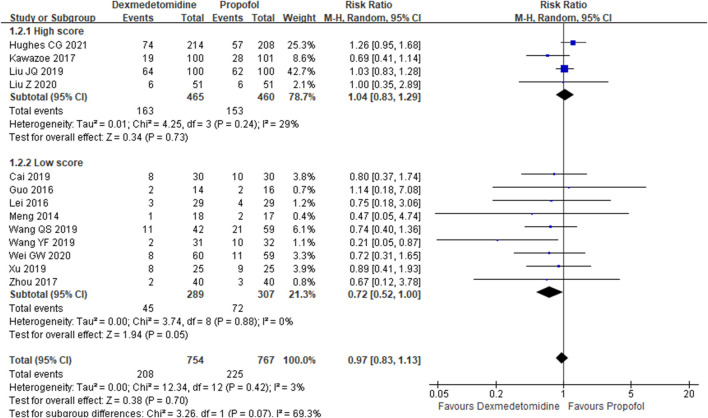
Forest plot of ICU stays.

**FIGURE 4:**
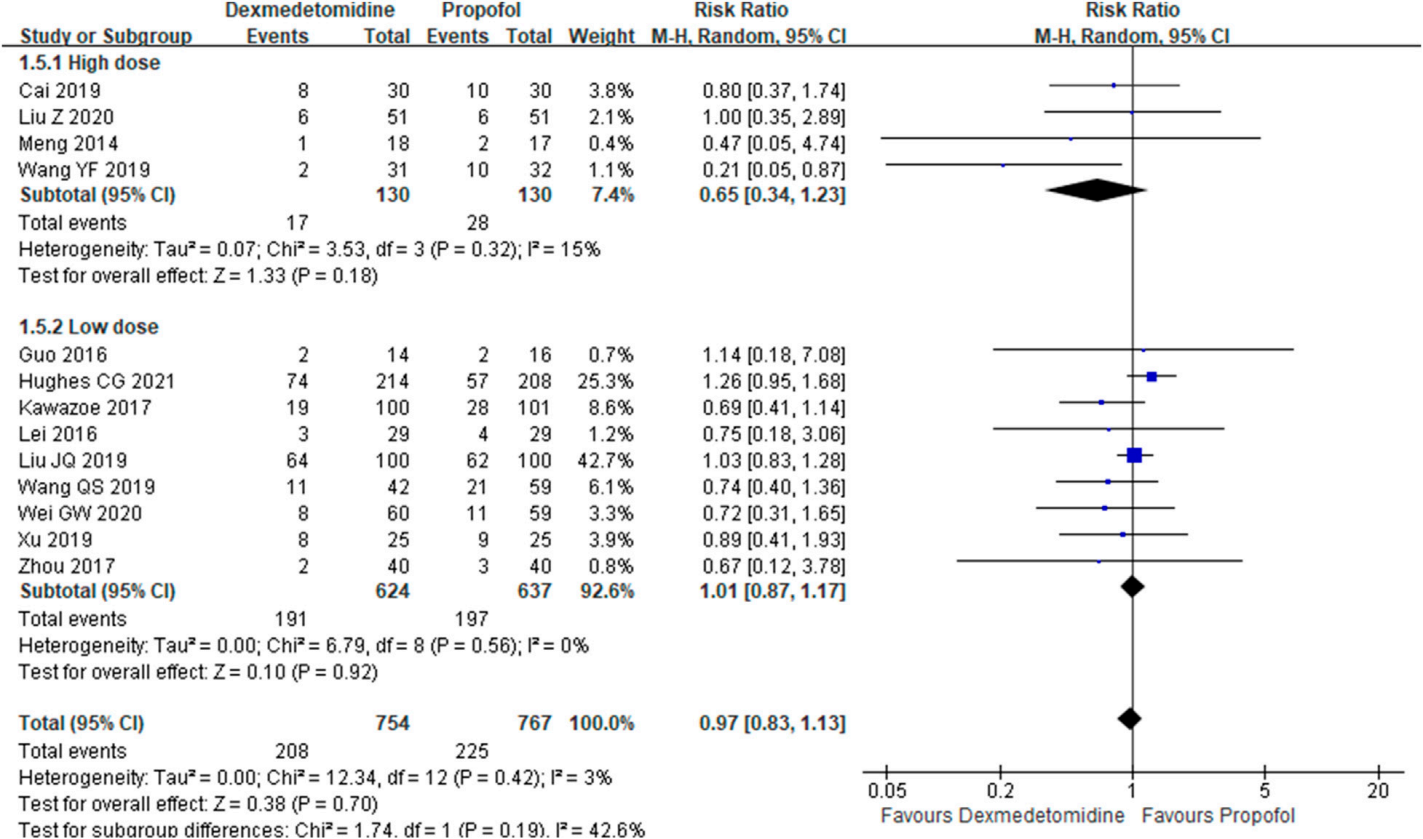
Forest plot of duration of mechanical ventilation.

The primary outcome reflected in the included studies is the 28-day mortality. A total of 13 studies (Meng, et al., 2014; Guo, et al., 2016; Lei and Li, 2016; Kawazoe, et al., 2017; Zhou, 2017; Cai, et al., 2019; Wang et al., 2019; Wang et al., 2019; Xu, 2019; Guowen and Bingyi, 2020; Liu et al., 2020; Zhan et al., 2020; Hughes Christopher et al., 2021) reported 28-day mortality according to the cases of 1,521 participants. Then, random effect models were utilized. Subgroup analysis was conducted according to studies published in English and non-English, high scores of Jadad and low scores of Jadad, high doses of DEX (≥0.5 μg/kg/h) and low doses of DEX. The result showed that there was no difference between the DEX group and propofol group in all the three subgroups (Figures 2–4).

### Secondary Outcomes

#### ICU Stays

**FIGURE 5:**
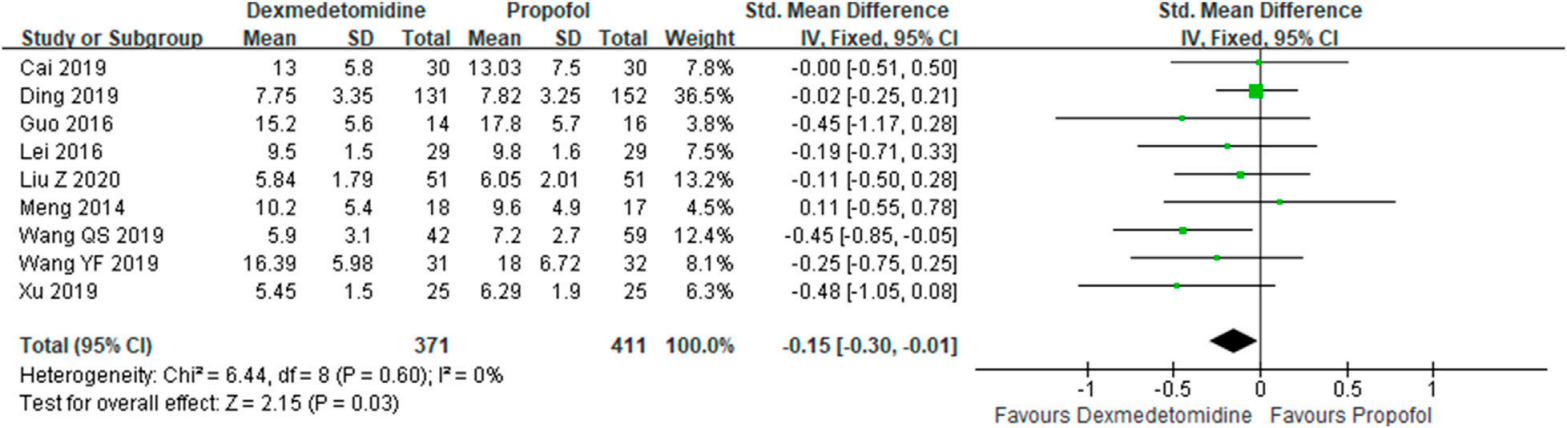
Forest plot of Incidence of adverse events.

A total of 10 studies (Meng et al., 2014; Guo, et al., 2016; Lei and Li, 2016; Zhou, 2017; Cai, et al., 2019; Ding, et al., 2019; Wang et al., 2019; Wang et al., 2019; Xu, 2019; Zhan et al., 2020) employed ICU stays as the evaluation index. The results indicated that the DEX group could reduce ICU stays in comparison with the propofol group {SMD: −0.15; 95% CI: [−0.30–(−0.01), *p* = 0.03]} (Figure 5).

### Duration of Mechanical Ventilation

There were six studies (Meng, et al., 2014; Guo, et al., 2016; Wang et al., 2019; Xu, 2019; Cai, et al., 2019; Zhan et al., 2020) which reported the duration of mechanical ventilation. We selected the fixed effect model since there was no heterogeneity in both the subgroups (I^2^ = 9.1%). Also, the meta-analysis showed that compared with propofol, DEX could reduce the duration of mechanical ventilation {SMD: −0.22; 95% CI: [−0.44-(−0.01), *p* = 0.043]} (Supplementary Figure S1).

### Incidence of Adverse Events

Seven studies (Kawazoe et al., 2017; Cai et al., 2019; Ding et al., 2019; Liu et al., 2019; Wang et al., 2019; Wang et al., 2019; Guowen and Bingyi, 2020) recorded the incidence of adverse events, which was evidenced by 889 participants. The results demonstrated that there was no difference in incidence of adverse events between the group of DEX and propofol [RR = 0.64, 95% CI = (0.37,1.11), *p* = 0.11] (Supplementary Figure S2).

### SOFA

Three studies (Cai, et al., 2019; Liu et al., 2019; Zhan et al., 2020) with 225 participants reported SOFA. The results demonstrated that SOFA decreased in the group of DEX in comparison with the group of propofol {SMD: −0.41; 95% CI: [−0.73–(−0.09), *p* = 0.013]} (Supplementary Figure S3).

#### Levels of IL-6 at 24 h and Levels of CK-MB at 72 h

Random effect models were utilized (I^2^ > 75%) in the above two outcomes. The results showed that there was no influence on levels of IL-6 at 24 h in the group of DEX in comparison with that in the group of propofol (SMD: −2.53; 95% CI: [−5.30–0.24], *p* = 0.074) (Supplementary Figure S4). The levels of CK-MB at 72 h decreased in the group of DEX in comparison with that in the group of propofol {SMD: −0.45; 95% CI: [−0.83– (−0.08), *p* = 0.017]} (Supplementary Figure S5).

## Discussion

### Findings

The analysis aims to access the efficacy and safety of DEX in patients with sepsis with the treatment of mechanical ventilation. The results showed that for patients with sepsis, the application of DEX had no advantage (28-day survival) compared with that of propofol. However, our analysis found that the use of DEX could decrease ICU stays, duration of mechanical ventilation, incidence of adverse events, SOFA, and levels of CK-MB at 72 h except the level of IL-6 at 24 h (Table 2). Thus, based on our analysis, this kind of an important outcome is supposed to be investigated further.

**TABLE 2:** Summary of meta-analysis.

Outcomes	Subgroup	No. of studies	No. of participants	Effect size (95% CI)	*p*
28-day mortality	non-English	10	698	RR, 0.74 (0.54, 1.02)	0.06
	English	3	823	RR, 1.03 (0.79, 1.34)	0.83
	Overall	13	1,521	RR, 0.97 (0.83, 1.13)	0.70
	Low score	9	596	RR, 0.72 (0.52, 1.00)	0.05
	High score	4	925	RR, 1.04 (0.83, 1.29)	0.73
	Overall	13	1,521	RR, 0.97 (0.83, 1.13)	0.70
	High dose	4	260	RR, 0.65 (0.34, 1.23)	0.18
	Low dose	9	1,261	RR, 1.01 (0.87, 1.17)	0.92
	Overall	12	1,521	RR, 0.97 (0.83, 1.13)	0.70
ICU stays	NA	10	966	SMD, -0.16 (-0.29, -0.02)	0.03
Duration of mechanical ventilation	NA	6	345	SMD, −0.22 (−0.44, −0.01)	0.043
Incidence of adverse events	NA	7	889	RR, 0.64 (0.37, 1.11)	0.11
SOFA	NA	3	225	SMD, −0.41 (-0.73, -0.09)	0.013
Levels of IL-6 at 24 h	NA	3	191	SMD, −2.53 (−5.30, 0.24)	0.074
Levels of CK-MB at 72 h	NA	3	420	SMD, −0.45 (−0.83, −0.08)	0.017

Note: SOFA, sequential organ failure assessment; RR, relative risks; WMD, weighted mean difference.

### Analysis

Compared with propofol, DEX has been reported to improve patient’s ability to communicate pain (Senem et al., 2020). As is known to all, in 2010, a significant randomized controlled trial named MENDS was conducted by Pandharipande PP, et al., which showed that DEX could reduce 28-day mortality in patients with sepsis, compared with those receiving lorazepam (Pandharipande Pratik et al., 2010). This result has brought great interest to researchers. Since then, a large number of studies have been carried out on the treatment of patients suffering from sepsis with dexmedetomidine. In 2017, Kawazoe et al. (2017) conducted a randomized controlled study (DESIRE) to evaluate the efficacy and safety of esmolol in septic shock. The results showed that dexmedetomidine did not obtain statistical significance in mortality. This is a negative result which may affect the use of dexmedetomidine in sepsis. However, we found that although there was no statistical significance, the study may have identified a clinically important advantage of dexmedetomidinean 8% reduction in 28-day mortality. Most randomized controlled trials select 28-day mortality as the primary outcome. In fact, long-term outcomes are very important in the research of sepsis. Until 2021, the latest research conducted by Hughes CG et al. (Hughes Christopher et al., 2021) selected 28-day mortality and 90-day mortality as the survival outcomes. The results said that for mechanically ventilated adult patients with sepsis, DEX did not decrease 90-day mortality in comparison with propofol (38 vs. 39%).

With respect to 28-day mortality, why are results of some studies negative and our results of meta-analysis positive? First, the sample size in most of the included studies is small, which limited the statistical power. For example, the DESIRE study showed a tendency to decrease 28-day mortality, and it is possible that the increase of the sample size would yield positive results. Second, the severity of patients with sepsis included in each study was different, and the therapeutic effect of DEX was also different. For example, the results from DESIRE studies demonstrated that DEX could reduce 28-day mortality (HR 0.39; 95%CI: 0.16–0.91; *p* = 0.03) for sepsis patients with APACHE Ⅱ ≥23. Therefore, with the deepening of relevant researchers, subgroup analysis of sepsis severity can be carried out in the future to further determine the appropriate population of DEX.

Why is dexmedetomidine beneficial for sepsis? According to the pharmacological mechanism of DEX, it is characterized by sedative and analgesic effects on the nerve activity as well as the inhibitory effect on the sympathetic nerve by activating the α2 receptor (Mohammed et al., 2020). Recently, more and more attention has been paid to the research on organ damage related to sepsis. The heart is one of the organs most frequently damaged by sepsis. The pathogenesis of sepsis cardiac dysfunction is varied, and mitochondrial damage is one of the important mechanisms (Yang and Zhang, 2021). Thus, the mechanism of DEX may manifest that the adrenergic pathway is activated by the α2 receptor, accelerating the metabolism and production of glucose in the body, replenishing and reconstructing damaged mitochondria in time, so as to relieve patient’s pain and anxiety as a way to protect their myocardial function.

We know that DEX could reduce the high heart rate, and based on the results, we found that DEX could reduce CK-MB levels. This finding indirectly suggests that DEX indeed has a protective effect on cardiac functions with the mechanism of inhibiting excessive sympathetic response, reducing myocardial oxygen consumption, alleviating myocardial mitochondrial damage, and improving the energy metabolism. Of course, more investigation on its mechanism remains to be launched.

Surprisingly, research studies on the effects of dexmedetomidine other than sedation have also been fruitful. Existing research results have shown that the non-sedative effects of dexmedetomidine mainly include anti-inflammatory and organ protection in an efficient way (Mohammed et al., 2020). The mechanism of action may be related to the activation of the cholinergic anti-inflammatory pathway as well as the cell layer, which needs further confirmation. In addition, our analysis did not demonstrate that DEX reduces IL-6 levels in comparison with propofol. Therefore, DEX may not offer advantages over propofol in terms of anti-inflammatory functions.

### Strengths and Limitations

Although two similar reviews have been published (Gao et al., 2019; Chen et al., 2020), there are some differences between our review and the two exiting reviews. First, most of the studies were not included in the exiting two reviews, which became an obstacle to the credibility of the results. Second, our review covers the largest number of studies.

There are several limitations in our meta-analysis. First, only four studies (Kawazoe et al., 2017; Ding et al., 2019; Liu et al., 2020; Hughes Christopher et al., 2021) were published in English. This would limit the extrapolation of results. Second, the sample size is so small in some of the included studies that the statistical power is limited. Third, since the sepsis patients received the comprehensive intervention, the influence of other united medications cannot be excluded.

## Conclusions

This meta-analysis suggests that the 28-day mortality in sepsis patients with the treatment of dexmedetomidine did not differ from those who received propofol. Besides, more high-quality trials are needed to confirm these findings.

## Data Availability

The original contributions presented in the study are included in the article/Supplementary Materials, and further inquiries can be directed to the corresponding authors.
