# Supplementary material for: Dexmedetomidine Versus Propofol for Patients With Sepsis Requiring Mechanical Ventilation: A Systematic Review and Meta-Analysis
Source: Front Pharmacol. 2021 Oct 14;12:717023. doi: 10.3389/fphar.2021.717023 (PMC8551708; doi:10.3389/fphar.2021.717023)
Supplement: Supplementary file 1 [file Table1.docx]

**Table S1 The quality of included studies**

| Study | The generation of random sequences | Allocation concealment | Blinding method | Reasons for withdrawal and dropout | Total scores |
| --- | --- | --- | --- | --- | --- |
| Meng et al, 2014 | 2 | 1 | 0 | 1 | 4 |
| Guo et al, 2016 | 2 | 1 | 0 | 1 | 4 |
| Lei et al, 2016 | 2 | 1 | 0 | 1 | 4 |
| Zhou, 2017 | 2 | 1 | 0 | 1 | 4 |
| Kawazoe et al, 2017 | 2 | 2 | 2 | 1 | 7 |
| Ding et al, 2019 | 2 | 1 | 2 | 1 | 6 |
| Liu JQ et al, 2019 | 2 | 1 | 2 | 1 | 6 |
| Wang QS et al, 2019 | 2 | 1 | 0 | 1 | 4 |
| Wang YF et al, 2019 | 2 | 1 | 0 | 1 | 4 |
| Liu SC et al, 2019 | 2 | 1 | 0 | 1 | 4 |
| Xu, 2019 | 2 | 1 | 0 | 1 | 4 |
| Cai et al, 2019 | 2 | 1 | 0 | 1 | 4 |
| Liu Z et al. 2020 | 2 | 2 | 0 | 1 | 5 |
| Wei GW et al, 2020 | 2 | 1 | 0 | 1 | 4 |
| Hughes CG 2021 | 2 | 2 | 2 | 1 | 7 |
