# Supplementary figures and images for: Dexmedetomidine Versus Propofol for Patients With Sepsis Requiring Mechanical Ventilation: A Systematic Review and Meta-Analysis
Source: Front Pharmacol. 2021 Oct 14;12:717023. doi: 10.3389/fphar.2021.717023 (PMC8551708; doi:10.3389/fphar.2021.717023)

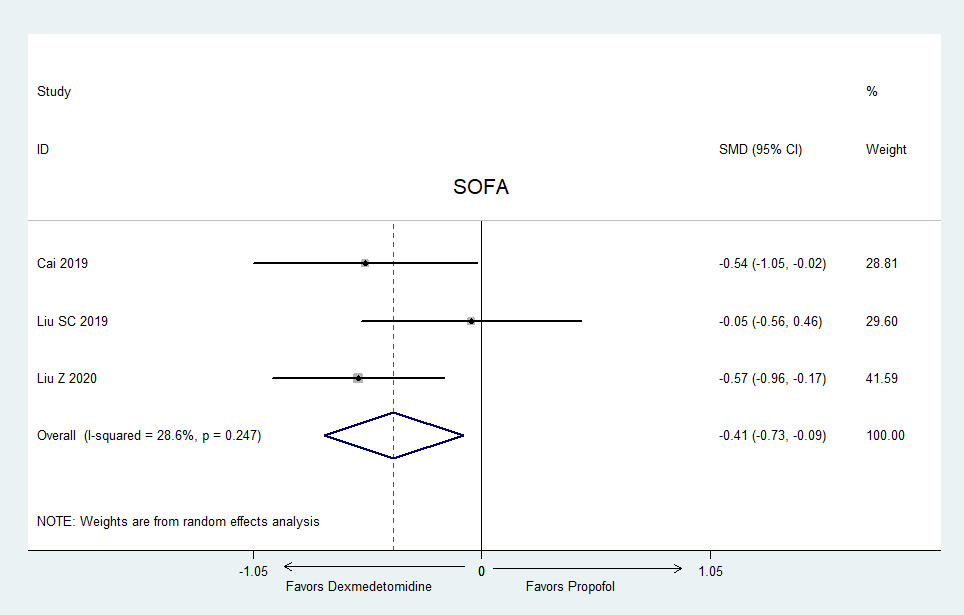

Supplement: Supplementary file 2 [file Image3.TIF]

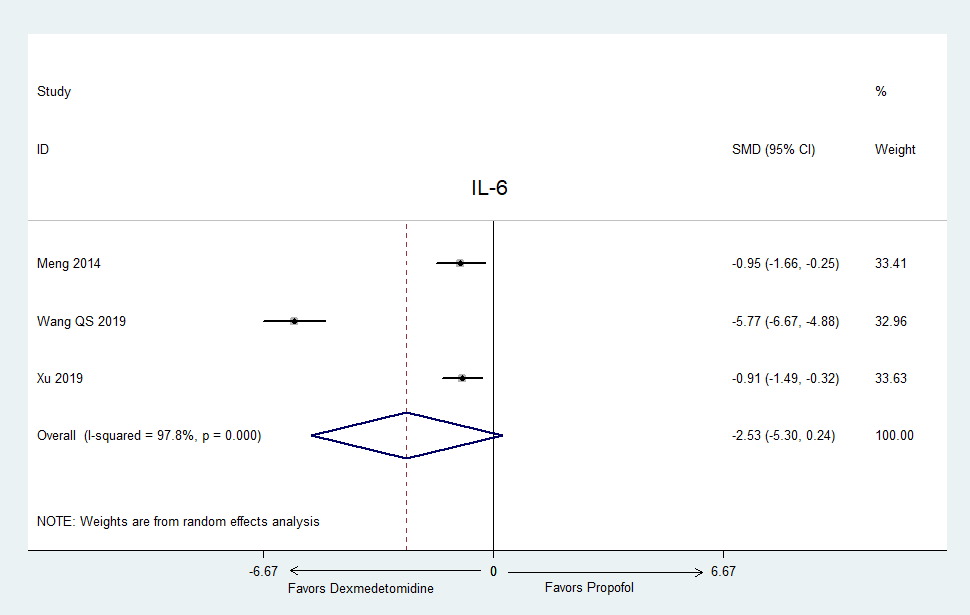

Supplement: Supplementary file 3 [file Image4.TIF]

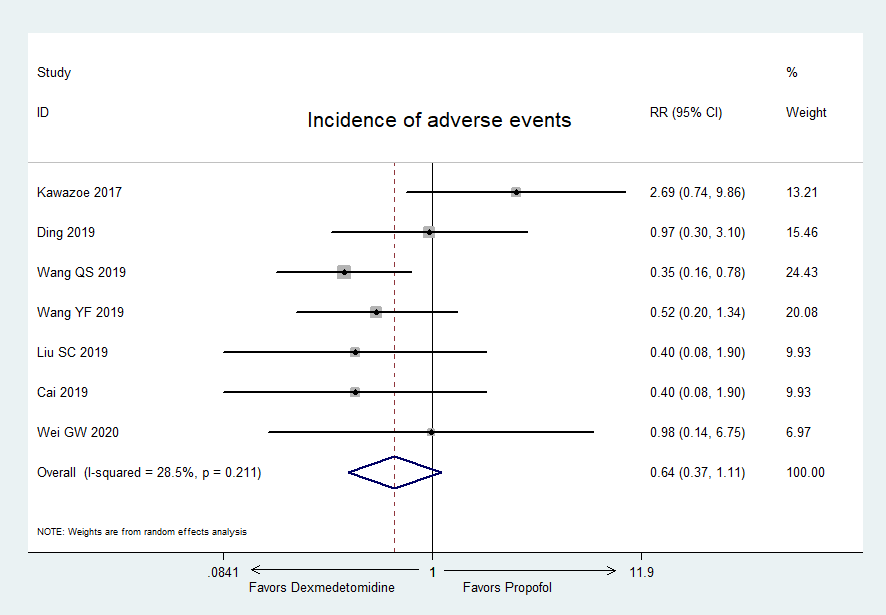

Supplement: Supplementary file 4 [file Image2.TIF]

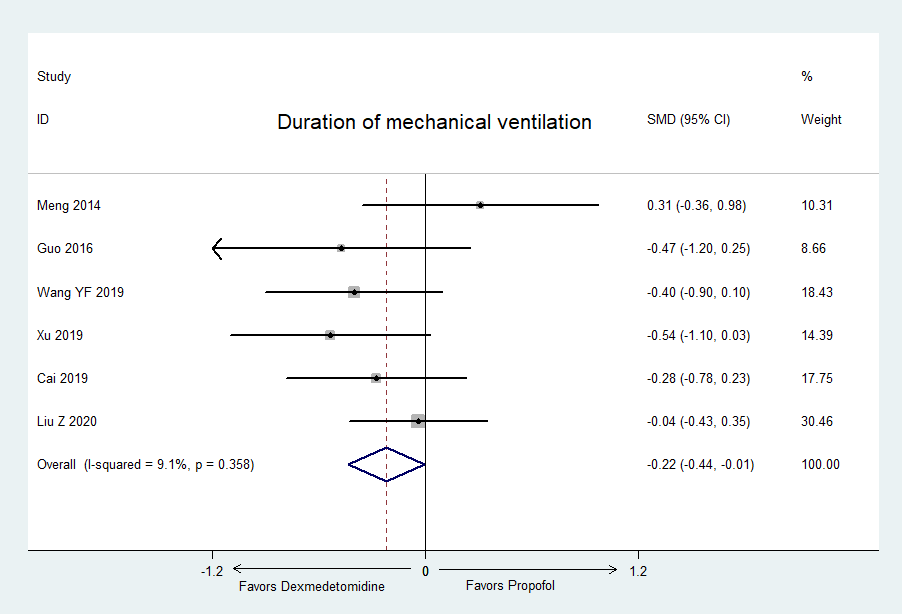

Supplement: Supplementary file 5 [file Image1.TIF]

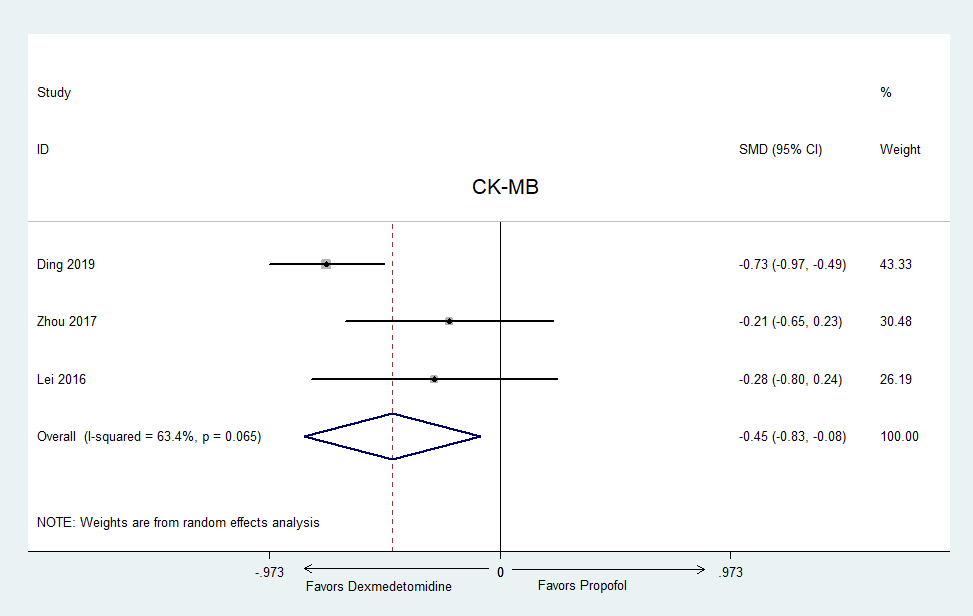

Supplement: Supplementary file 6 [file Image5.TIF]
